# Ubiquitination of MAP1LC3B by pVHL is associated with autophagy and cell death in renal cell carcinoma

**DOI:** 10.1038/s41419-019-1520-6

**Published:** 2019-03-22

**Authors:** Hyun Mi Kang, Kyung Hee Noh, Tae Kyung Chang, Dongmin Park, Hyun-Soo Cho, Jung Hwa Lim, Cho-Rok Jung

**Affiliations:** 1 Korea Research Institute of Bioscience and Biotechnology (KRIBB), 125 Gwahak-ro, Daejeon, Republic of Korea; 2 Department of Functional Genomics, Korea University of Science and Technology (UST), 217 Gajeong-ro, Daejeon, Republic of Korea

## Abstract

Von Hippel Lindau (VHL) expression is significantly decreased in high-grade RCC, and autophagy, which is involved in tumor growth, invasion, differentiation, and metastasis, is activated in various human cancers. However, the relationship of autophagy and VHL in tumor progression remains controversial. Here, we showed that the expression levels of VHL and microtubule-associated protein 1 light chain 3B (MAP1LC3B, LC3B) were inversely correlated with various tumor grades of RCC tissues. pVHL was found to possess the LIR motif within a beta domain that interacted with MAP1LC3B and ubiquitinated it. The L101A VHL mutant failed to interact with MAP1LC3B, thereby failing to induce ubiquitination. MAP1LC3B-mediated autophagy was inhibited by functional pVHL and the ubiquitination of MAPLC3B was implicated in autophagy-induced cell death. We screened various autophagy inducers to determine the physiological function of the inhibition of LC3B-mediated autophagy by pVHL using VHL-deficient and VHL-expressing cell lines. MLN9708, a proteasome inhibitor, potently induced autophagy via the induction of MAP1LC3B and sensitized the cell to autophagy-mediated cell death in VHL-deficient and VHL-mutant (L101A) cells. In conclusion, our results showed that pVHL interacts with MAPL1LC3B and inhibits LC3B-mediated autophagy via MAP1LC3B ubiquitination. Furthermore, the activation of autophagy by the proteasome inhibitor MLN9708 induced cell death, indicating that MLN9708 can be used for VHL-deficient RCC therapy.

## Introduction

Autophagy is important for maintaining cell homeostasis as it removes damaged intracellular organelles or abnormal proteins. In addition to these basic functions, autophagy is involved in various physiological and pathological phenomena. Autophagy is induced when cells are exposed to stressful environmental conditions, such as nutrient depletion or infection, to regulate cell growth and death^1^. The function of autophagy depends on the cellular context. In cancer cells, autophagy is involved in suppression of tumorigenesis. This is because beclin 1 (*BECN1*), which is important for autophagy induction, is defective in breast and ovarian cancers^2^. Indeed, induction of BECN1 in MCF7 breast cancer cells induced autophagy and inhibited proliferation of cancer cells^2^. However, other reports showed that autophagy is upregulated in the late stage of cancer^3,4^. Autophagy induced cell death-independent apoptosis in various types of cancer^5^. Tamoxifen, which targets the estrogen receptor, induces autophagic cell death, and temozolomide, a DNA alkylation agent, also induces autophagic cell death in malignant glioma cells^6,7^.

The von Hippel-Lindau (*VHL*) tumor suppressor encodes the VHL protein, which targets hypoxia inducible factor 1-alpha (HIF-1α)^8^. In addition, VHL is involved in the assembly of the extracellular matrix and regulation of microtubule stability and mitochondrial function^9–11^. Loss of VHL function frequently occurs in renal cell carcinoma (RCC) and its tumorigenicity is suppressed by gain of VHL function^12^. VHL-deficient RCC is aggressive and chemoresistant due to activation of HIF-2 α^13^ but is sensitive to autophagic cell death^14^. Autophagic cell death is one of the therapeutic options for VHL-deficient RCC, although the mechanism via which VHL regulates autophagy is still not known. In this study, we showed that VHL targets microtubule-associated protein 1 light chain 3B (MAP1LC3B) and downregulates autophagy. Furthermore, we observed that the proteasome inhibitor, MNL9708, induces cell death in VHL-deficient cells both in vitro and in vivo.

## Results

### Relevant clinical data and diagnosis

**Fig. 1:**
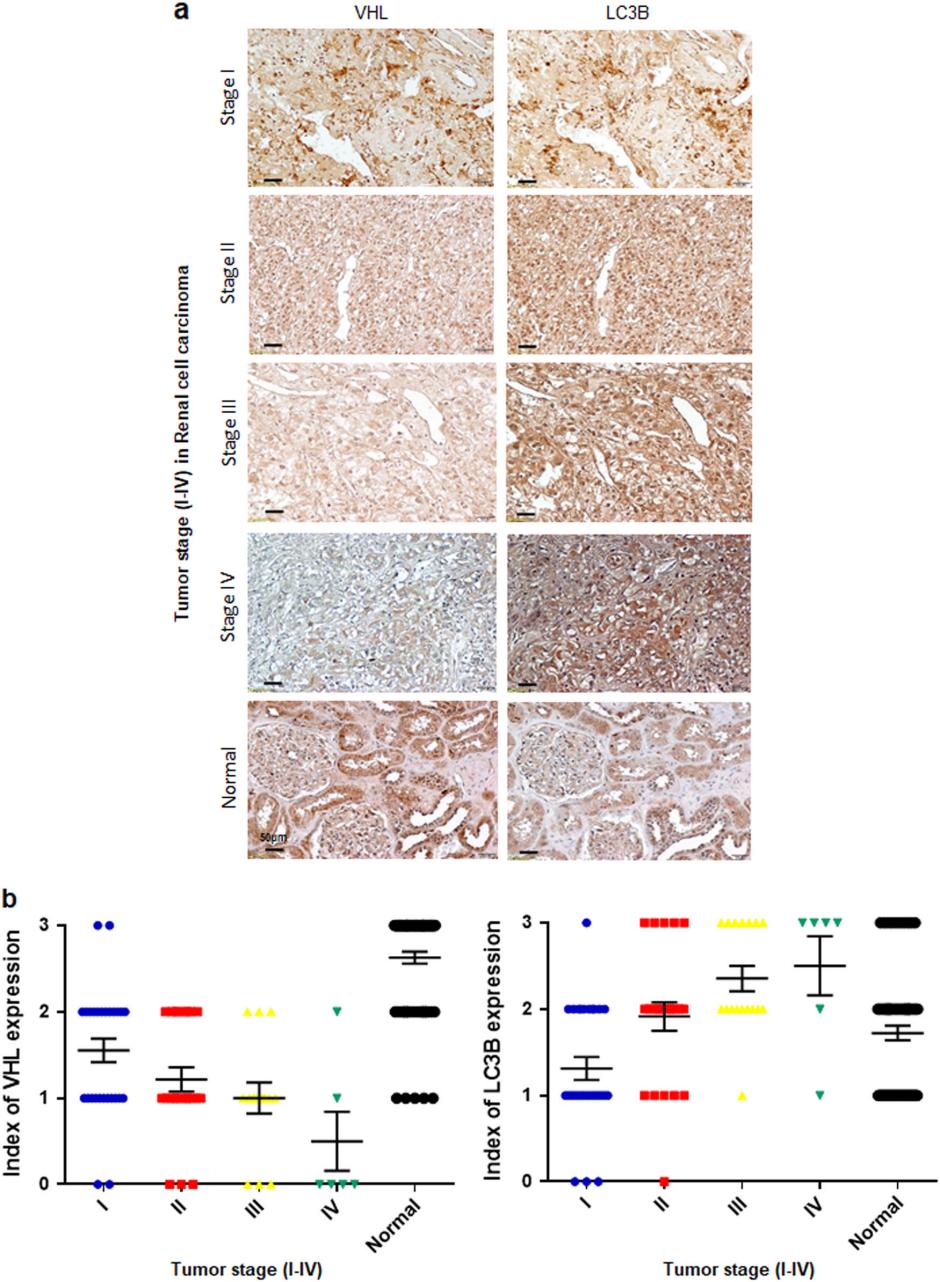
Association of VHL or LC3B expression with clinical significance in patients with RCC. **a** Representative images of immunohistochemical staining of VHL and LC3B in samples from RCC patients at different TNM stages. **b** Quantification of VHL and LC3B staining score in RCC patients at different TNM stages

VHL is mutated or silenced in >50% cases of sporadic clear cell renal cell carcinomas (ccRCC), and autophagy is observed in most cancer cells. To identify the correlation between VHL and LC3B expression in human RCC tissues, a tissue microarray (TMA) generated from 144 ccRCC and 78 normal kidney tissues was subjected to immunohistochemical analysis using DAB (3,3–diaminobenzidine) staining. VHL expression was significantly lower in RCC patients with a higher tumor grade, whereas LC3B expression was significantly higher in RCC patients with a higher tumor grade. Thus, VHL and LC3B expression levels were inversely correlated (immunohistochemistry, *P* < 0.01; Fig. 1a, b). These results indicated that autophagy activation induces tumor progression in VHL-deficient RCC.

### VHL inhibits LC3B–mediated autophagy

**Fig. 2:**
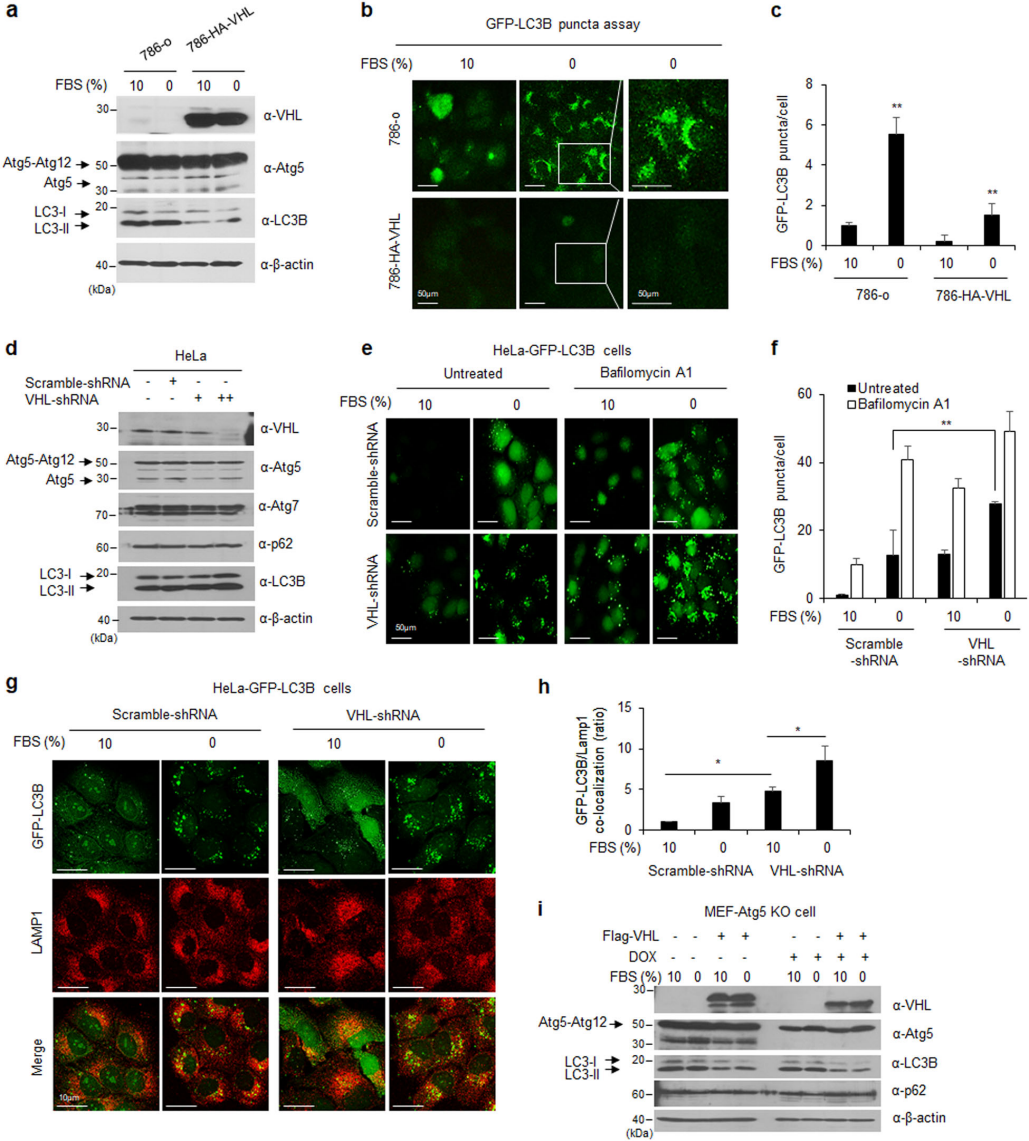
Regulation of autophagy signal by VHL expression. **a** The 786-o or 786- HA-VHL cells were cultured in complete media with 10% FBS or serum-free media for 24 h and analyzed using western blotting. **b** The 786-o or 786-HA-VHL cells were transfected with 10 µg GFP-tagged LC3B plasmid, cultured under the same conditions as in Fig. 2a, and observed using a fluorescence microscope. **c** The GFP-LC3B puncta per cell (*N* = 10/group) were quantified using the ImageJ software. **d** HeLa cells were transfected with 15 µg scrambled shRNA or 5 and 15 µg VHL-shRNA plasmid. After transfection for 48 h, the cells were analyzed using western blotting with the indicated antibodies. **e** After transfection of HeLa cells stably expressing GFP-LC3B with scrambled shRNA or VHL-shRNA plasmids, the cells were cultured in complete media (10% FBS) or serum-free media (0% FBS) with or without 10 µM bafilomycin A1 treatment for 24 h. Subsequently, the cells were observed using fluorescence microscopy. **f** The puncta in the representative image were quantified using the ImageJ software. **g** HeLa cells stably expressing GFP-LC3B were transfected with scrambled shRNA or VHL-shRNA plasmids, stained with anti-LAMP1 (used as a lysosome marker), and observed using confocal microscopy. DAPI was used for nuclear staining. **h** The representative image was quantified using the ImageJ software. Error bars indicate the S.D. of means (*P-value < 0.05, **P-value < 0.01). **i** The *Atg5* knockout MEFs were either left untreated or were treated with 20 ng/ml doxycycline hydrochloride (DOX) for 5 days. The treated/not-treated *Atg5* KO MEFs were transfected with 15 µg Flag-VHL plasmid, cultured in complete medium with 10% FBS or serum-free DMEM for 24 h, and then analyzed using western blotting

To investigate autophagic regulation in VHL-deficient RCC cells, 786-o and RCC4 cells were cultured in complete medium with 10% FBS or serum-free medium without FBS for starvation-induced autophagy. The expression of LC3B, ATG5, and p62, the major initiation markers of autophagy, was analyzed using western blotting in various RCC cell lines with or without VHL (Fig. 2a and Supplementary Fig. 1a). Results show that overexpression of VHL did not affect the ATG5-ATG12 complexes but inhibited both the cytosolic (LC3B-I) and lipid-modified (LC3B-II) forms of LC3B. The LC3B-II form is involved in autophagosome membrane expansion and fusion and is used as an indicator of autophagy. The mRNA levels of *LC3* family genes and *ATG*-related genes in VHL-defective RCC cells or VHL- expressing cells were analyzed using reverse transcription-polymerase chain reaction (RT-PCR) and specific primers. The mRNA levels of autophagy-related genes were not affected in RCC cells with or without VHL expression under starvation-induced autophagy (Supplementary Fig. 1b). To confirm the functional relationship of VHL with inhibition of LC3B expression, the formation of LC3B puncta, which is a characteristic of autophagosome accumulation, was observed in 786-o or RCC4 cells using fluorescence (Fig. 2b and Supplementary Fig. 2). The intensity of LC3B punctate staining per cell was quantified using the ImageJ software (Fig. 2c). Results showed that VHL overexpression decreased the intensity of LC3B punctate, which is associated with downregulation of LC3B. VHL inhibited LC3B expression, indicating that VHL represses LC3B-mediated autophagosome formation. To confirm the VHL dependency of LC3B regulation, we artificially downregulated VHL expression in HeLa cells (expressing wild-type VHL) using specific VHL-shRNA and showed that LC3B expression is restored by knocking out VHL (Fig. 2d). To observe the formation of LC3B punctate after silencing VHL, we generated HeLa cells stably expressing GFP-LC3B, incubated the cells under starvation (0% FBS) or non-starvation conditions (10% FBS) and then treated them with bafilomycin A1. Untreated cells were used as the control. Bafilomycin A1 is widely used as an inhibitor of autolysosome fusion to determine autophagic flux. As expected, there were more GFP-LC3B punctate in cells with silenced VHL (HeLa-GFP-LC3B cells treated with VHL-shRNA) than in cells with wild-type VHL (treated with scrambled shRNA; Fig. 2e, f). To determine whether the LC3B punctate were active, we performed immunofluorescence staining using an antibody against lysosomal associated membrane protein 1 (LAMP1), a lysosomal marker, in 786-o or 786-HA-VHL cells and quantified the co-localization of LC3B-LAMP1 using the ImageJ software under each condition. The LC3B punctate structure induced by VHL deficiency co-localized with LAMP1, indicating the fusion of the autophagosome with lysosome (Fig. 2g, h). To confirm whether VHL inhibited LC3B-mediated autophagy, wild-type, and *Atg5* knockout mouse embryonic fibroblast cells were transfected with a VHL-expressing vector and cultured in the absence or presence of doxycycline. Subsequently, the cells were induced for autophagy through serum starvation and the expression of autophagy-related genes was analyzed using western blotting. Results showed that the reduction of LC3B expression by VHL was independent of its association with Atg5 expression (Fig. 2i). These results suggested that VHL regulated LC3B-mediated autophagy in RCC cells.

### VHL directly binds to LC3B, the major marker of autophagy

**Fig. 3:**
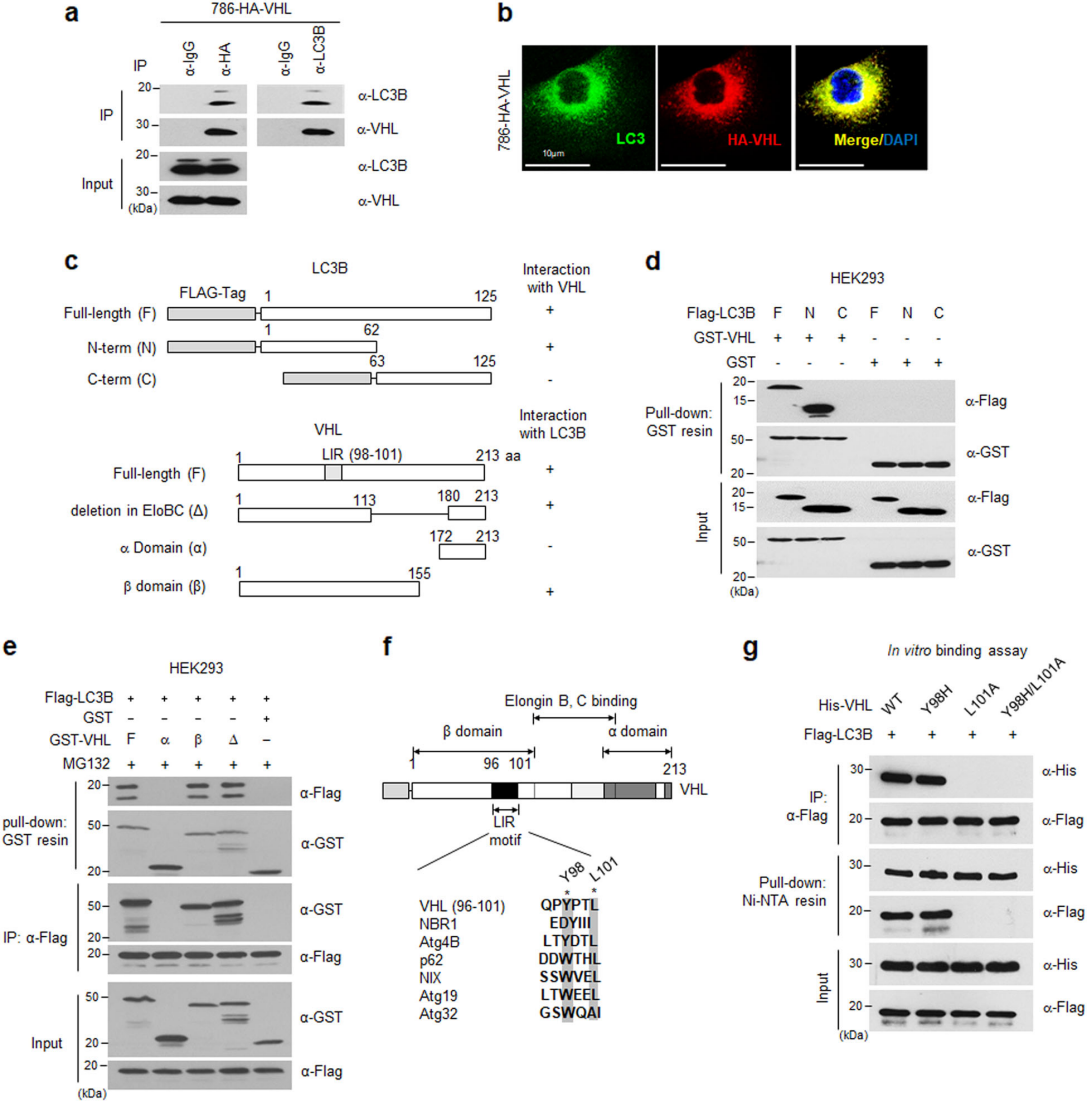
Identification of the LIR motif of VHL required for interaction with the N-terminus of LC3B. **a** Interaction of endogenous VHL and LC3B was assessed using immunoprecipitation assay and analyzed using western blotting with the indicated antibodies. **b** For detecting co-localization of endogenous VHL and LC3B, cells were stained with anti-LC3B (FITC-conjugated anti rabbit-IgG, green) and anti-VHL (rhodamine-conjugated anti mouse-IgG, red), and observed using confocal microscopy. **c** Schematic of different LC3B (top) and VHL (bottom) mutants. **d** HEK293 cells were transfected with 5 µg each of wild-type Flag-LC3B (F; 1–125 amino acids) or its various mutants (N; 1−62 amino acids or C; 63−125 amino acids) and 5 µg of GST-VHL or GST plasmids. At 24 h after transfection, the cells were pulled down with GST-Sepharose resin and analyzed using western blotting. **e** HEK293 cells were transfected with 5 µg each of wild-type GST-VHL (F; 1−213 amino acids) or its various mutants (α; 172−213 amino acids, α-domain; β; 1−155 amino acids, β-domain; Δ; 114−179 amino acid deletion, deletion of elongin B/c bind site) and 5 µg GST-VHL or GST plasmids for 24 h. Next, the cells were harvested, pulled down with GST resin, and analyzed using western blotting. **f** Schematic of the LIR motif of VHL and its various mutants. **g** Wild-type His-VHL or fusion proteins of its various mutants were expressed and purified from *Escherichia coli*. Flag-tagged LC3B was purified using anti-Flag agarose bead in Flag-LC3B overexpressing HEK293 cells. Each indicated protein (1 µg) was incubated at 4 °C overnight, immunoprecipitated with an anti-Flag antibody or pulled down with Ni-NTA resin and analyzed using western blotting with the indicated antibodies

To further investigate regulation of LC3B-mediated autophagy by VHL, we performed an immunoprecipitation assay with anti-HA or anti-LC3B in 786-HA-VHL cells. Anti-IgG was used as a negative control for immunoprecipitation (Fig. 3a). Endogenous LC3B interacted with HA-VHL. To determine whether the endogenous LC3B proteins co-localized with VHL, GFP-tagged LC3B was transiently expressed in 786-HA-VHL cells. We observed that LC3B co-localized with VHL in the cytosol (Fig. 3b). To determine the region of LC3B that binds to VHL, various truncations of LC3B were generated based on the sequence of the N-terminally Flag-tagged wild-type LC3B. Truncated mutants of GST-tagged VHL have been previously reported^15^ (Fig. 3c). HEK293 cells were transfected with the indicated plasmids, the VHL complexes were immunoprecipitated using glutathione Sepharose beads, and the precipitate was analyzed using western blotting. Results showed that the wild-type VHL binds with the Flag-tagged wild-type and N-terminus, but not the C-terminus of LC3B. During autophagosome formation, LC3 proteins (LC3-I) are processed at the C-terminus and the residual N-terminus is conjugated with phosphatidylethanolamine (PE, these processed proteins are called LC3-II), which fuses with the autophagosome membrane. Results showed that VHL binds to both LC3-I and LC3-II, which are involved in autophagosome formation (Fig. 3d). In addition, wild-type LC3B binds to the β-domain of VHL, and the elongin-binding domain of VHL did not affect interaction with LC3B (Fig. 3e). Next, to identify specific regions in VHL that bind to LC3B, we analyzed VHL protein sequences using the iLIR software, used for predicting the LC3 interacting region (LIR) motif. Most LC3 binding proteins harbor the LIR motif. The LIR motif searching program revealed conserved LIR motif sequences in VHL (96−101 amino acids; Fig. 3f). To determine whether the LIR motif of VHL specifically binds to LC3B, we generated point mutants of the VHL LIR motif (VHL-Y98H; VHL-L101A; VHL-Y98H and L101A, a double point mutant containing Y98H and L101A) using site-directed mutagenesis. Wild-type or mutant VHL and Flag-tagged LC3B were expressed, purified from *Escherichia coli*, and used in in vitro binding assay with the indicated proteins. Results show that the L101A mutation completely prevented the interaction between VHL and LC3B, whereas the histidine mutation at tyrosine 98 had no effect (Fig. 3g). Similar results were obtained in an in vivo co-immunoprecipitation assay in HEK293 cells (Supplementary Fig. 3a). Taken together, the N-terminal LC3B and LIR motif of VHL interacted directly both in vitro and in vivo.

### LC3B is a novel substrate of the VHL E3 ubiquitin ligase

**Fig. 4:**
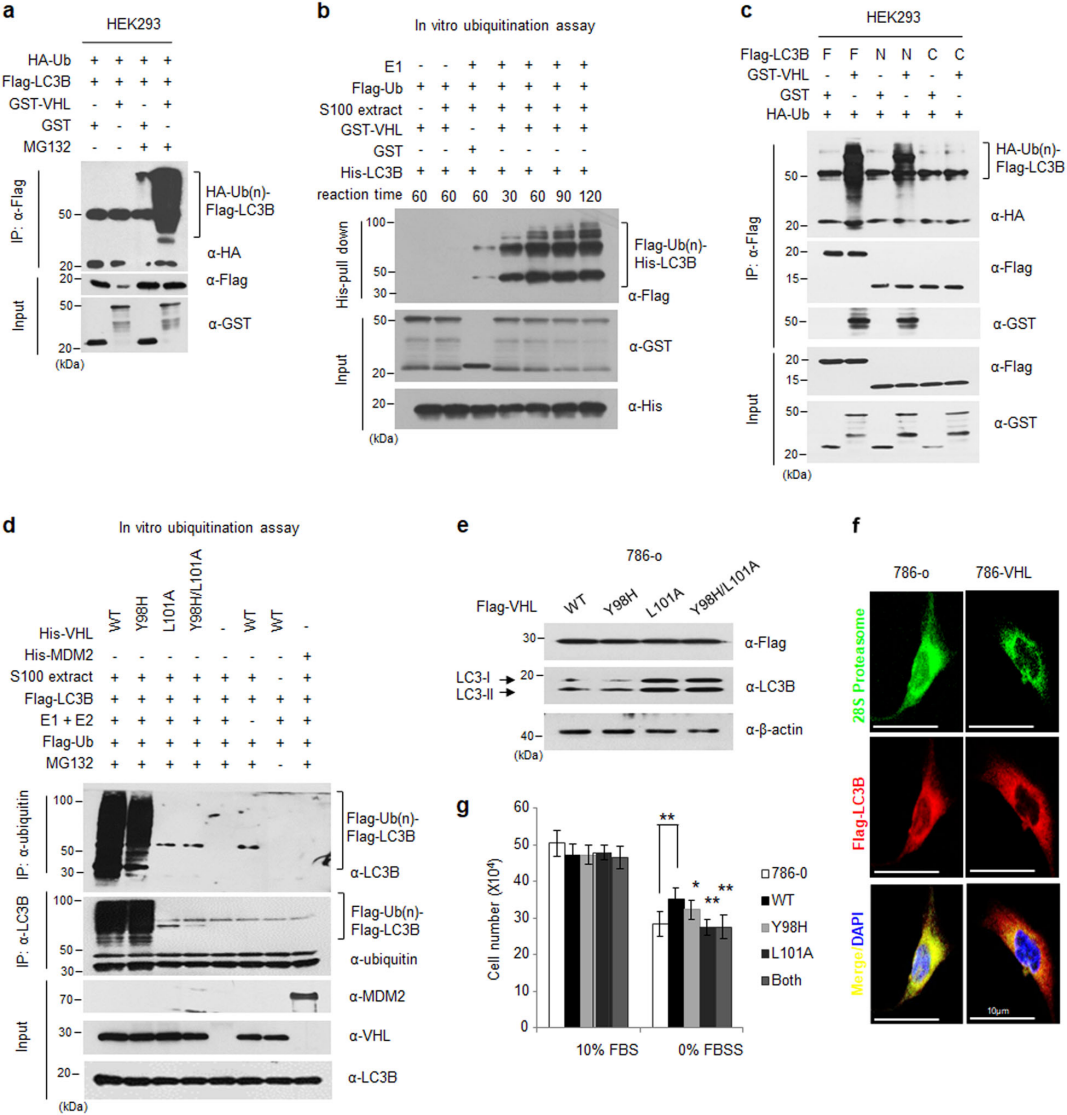
Polyubiquitination of LC3B is induced by the VHL E3 ubiquitin ligase. **a** HEK293 cells were transfected with 5 µg each of HA-Ub, Flag-LC3B, and GST-VHL/or GST plasmids. After transfection for 24 h, the cells were treated with 10 µM MG132 (a proteasome inhibitor) for 16 h. The cells were then immunoprecipitated with a Flag antibody and analyzed using western blotting. Western blot analysis showed polyubiquitination of LC3B in HEK293 cells expressing VHL. **b** The GST-VHL/ or GST and His-LC3B fusion proteins were expressed in *E. coli* and were purified as described in the Methods section. The purified proteins were incubated at 37 °C in the presence or absence of E1, ATP, and ubiquitin and were analyzed using western blotting. The S100 extract from 786-o cells is essential for purifying the VHL E3 ubiquitin ligase complex. **c** HEK293 cells were transfected with 5 µg each of Flag-LC3B or Flag-LC3B domain mutants, GST-VHL or GST, and HA-Ub plasmids. The cells were treated with MG132, immunoprecipitated with Flag antibody, and analyzed using western blotting. **d** Each protein was purified from *E. coli* (His-VHL, Hid-MDM2) or HEK293 cells (Flag-LC3B) and used in an in vitro ubiquitination assay with 0.5 µg wild-type VHL and its various mutants (Y98H, L101A, and Y98H/L101A mutants) and 5 µg Flag-LC3B. The reaction mixtures were immunoblotted as indicated. **e** The 786-o cells were transfected with 5 µg each of wild-type Flag-VHL and its various mutants for 24 h and analyzed using western blotting. **f** Co-localization of the 28 S proteasome and LC3B was determined using an immunofluorescence assay combined with confocal microscopy in 786-o or 786-HA-VHL cells. **g** The 786-o cells were transfected with 5 µg each of wild-type Flag-VHL and its various mutants for 24 h and then cultured in complete medium (10% FBS) or serum-free medium (0% FBS) for 24 h. The cultured cells were harvested and counted using a hematocytometer

We next investigated whether direct interaction between VHL and LC3B is related to functional activity of VHL as an E3 ubiquitin ligase. As VHL is known as an E3 ubiquitin ligase complex, we speculated that LC3B could be its specific novel substrate. To verify this hypothesis, GST-tagged VHL, Flag-tagged LC3B, and HA-tagged ubiquitin were transfected into HEK293 cells. To prevent proteasomal degradation of ubiquitinated proteins, the cells were treated with MG132, a proteasome inhibitor, for 16 h after transfection. As shown in Fig. 4a, Flag-tagged LC3B was immunoprecipitated with anti-Flag agarose. Similar to that observed in Fig. 3, LC3B bound to GST-tagged VHL and was polyubiquitinated with HA-Ub under the same conditions. LC3B protein level was lower without MG132 treatment than with MG132 treatment. To directly determine LC3B ubiquitination by VHL without interference from other cellular factors, GST-VHL and His-LC3B proteins were purified from *E. coli*. The E3 ligase activity of VHL protein and the S100 extract purified from *E. coli* or 786-o cells was identified using an in vitro HIF-ODD ubiquitination assay with a well-known HIF-ODD domain as the VHL substrate (Supplementary Fig. 3b). We performed an in vitro ubiquitination assay using each indicated protein and an ATP-regeneration system. We observed that LC3B was polyubiquitinated by the VHL E3 ubiquitin complex in a time-dependent manner (Fig. 4b) and was subsequently degraded via the proteasome. Next, we confirmed the region of LC3B ubiquitinated by VHL using the N-terminus and C-terminus LC3B mutants. Results showed that the full-length and N-terminal Flag-tagged LC3B were polyubiquitinated by GST-VHL but not the C-terminal LC3B alone. This indicated that E3 ubiquitin ligase complexes containing VHL can ubiquitinate the LC3B proteins, full-length as well as the C-terminal processed LC3B-II (Fig. 4c). To evaluate whether the LIR motif of VHL is required during LC3B ubiquitination, the in vitro ubiquitination assay was performed using VHL mutant proteins, VHL-Y98H, VHL-L101A, and VHL-Y98H/L101A and the ATP regeneration system. Flag-tagged LC3B was polyubiquitinated by wild-type and Y98H mutant VHL, but not mutant VHL contained the leucine 101 mutation (L101A and double point mutant; Fig. 4d). We next investigated whether LC3B protein level was affected by the VHL LIR motif mutants. VHL mutant plasmids were transfected into VHL-deficient 786-o cells, followed by analysis of LC3B protein levels using western blotting (Fig. 4e). As shown in Fig. 4e, cell lysates transfected with L101A VHL mutant did not affect LC3B degradation. Immunofluorescence staining was performed to identify the localization of LC3B and the 28S proteasome in cells with respect to VHL expression. Results show that Flag-LC3B and the 28S proteasome proteins co-localized more in VHL-deficient 786-o cells than in 786-o cells stably expressing VHL (Fig. 4f). This indicated that VHL ubiquitinated the *N*-terminal region (1−162 amino acids) of LC3B, and the leucine 101 in the LIR motif of VHL is essential for LC3B ubiquitination. To verify whether the ubiquitination of LC3B by VHL is involved in cell death, we analyzed the difference in the death rates of cells expressing wild-type and mutant VHL, which cannot interact with LC3B (L101A), in normal and/or serum depravation condition (Fig. 4g and Supplementary Fig. 4). The cells expressing the VHL mutant (VHL-L101A) were more sensitive to the serum depravation condition, similar to VHL-depleted cells. This result suggested that the modulation of LC3B level by VHL is involved in induction of cell death under stress.

### Autophagy inducing compounds induce cell death by enhancing autophagy and apoptosis signal in VHL-deficient RCC cells

**Fig. 5:**
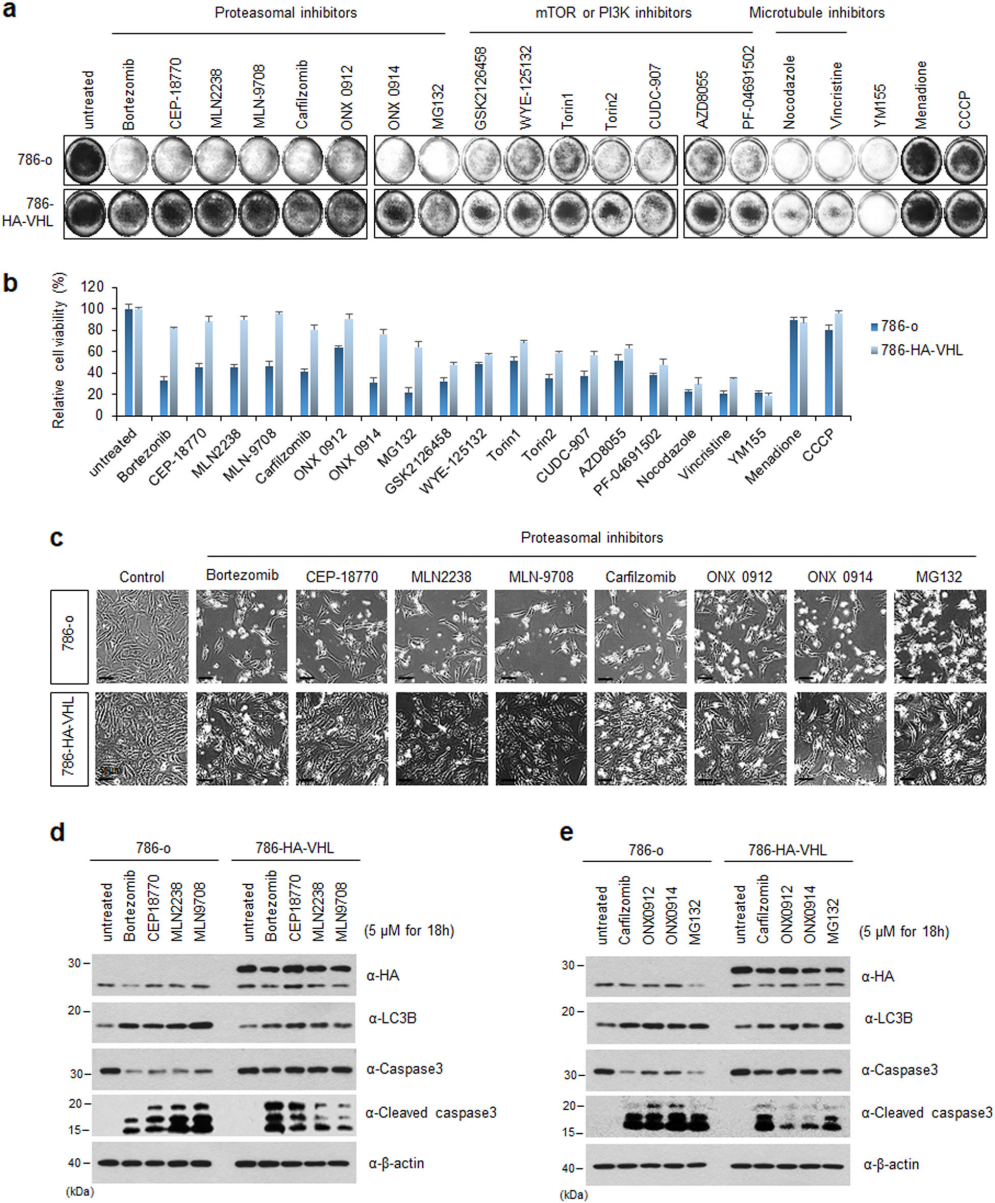
Effect of autophagy inducers on cell viability and autophagic marker expression in wild-type VHL and VHL-defective RCC cells. **a** The 786-o (VHL null cell line) or 786-o stably expressing wild-type VHL (786-HA-VHL) cells were tested for cell death effect by inducing hyper autophagic flux with a library of known autophagy modulators. The cells were treated with 5 µM of each chemical for 18 h and stained using crystal-violet. **b** After chemical treatment, the cells were incubated with WST-1 solution at 37 °C for 1 h, and absorbance at 450 nm was measured using a microplate reader. Error bars indicate standard deviation (S.D.) of means. **c** After treatment as mentioned in Fig. 1a, the cells were observed using phase contract microscopy. Scale bars represent 100 µm. **d**, **e** The 786-o and 786-HA-VHL cells were treated with 5 µM of each chemical for 18 h, and then analyzed using western blotting with the indicated antibodies

Dysregulation of autophagy due to less or excessive regulation disrupts the dynamic balance between proliferation and cell death and results in apoptosis. Therefore, we investigated whether induction of autophagy can sensitize VHL-deficient cells such as in RCC to apoptosis. Toward this objective, we screened 152 autophagy inducing compounds against an RCC cell line stably expressing wild-type VHL (786-HA-VHL) and VHL-deficient RCC (786-o) cells and evaluated cell death (data not shown) using crystal violet staining and the WST-1 assay. Seventeen autophagy inducers, such as mTOR or PI3K inhibitors, proteasome inhibitors, and microtubule inhibitors, increased cell death more in 786-o cells than in 786-HA-VHL cells (Fig. 5a, b). Among them, the proteasome inhibitors induced cell death more strongly in 786-o cells than in 786-HA-VHL cells after the treatment (Fig. 5c). The expression of the LC3B-II form was high in the 786-o cells treated with eight proteasome inhibitors, which is indicative of autophagy activation; this was further confirmed by the appearance of a cleaved form of caspase-3, an indicator of apoptosis, only in the 786-o cells and not in the 786-HA-VHL cells (Fig. 5d, e). To determine the type of cell death induced by these eight proteasomal inhibitors, we performed apoptosis cytofluorimetric assay combined with annexin V and 7-AAD activity assays. Results showed that all proteasome inhibitors that induced autophagy triggered apoptosis more strongly in 786-o cells than in 786-HA-VHL cells (Supplementary Fig. 5). As shown in Fig. 1, the level of the active autophagy-related protein, LC3B-II, was higher in 786-o cells than in 786-HA-VHL cells. Furthermore, we performed autophagy cytofluorimetric analysis using the anti-LC3 antibody on a Muse cell analyzer under the same conditions. The expression of LC3B was greater in 786-o cells than in 786-HA-VHL cells after proteasome inhibitor treatment (Supplementary Fig. 6).

### Proteasome inhibitor MLN9708 suppresses tumor progression of VHL-deficient RCC cells via induction of hyper-autophagy

**Fig. 6:**
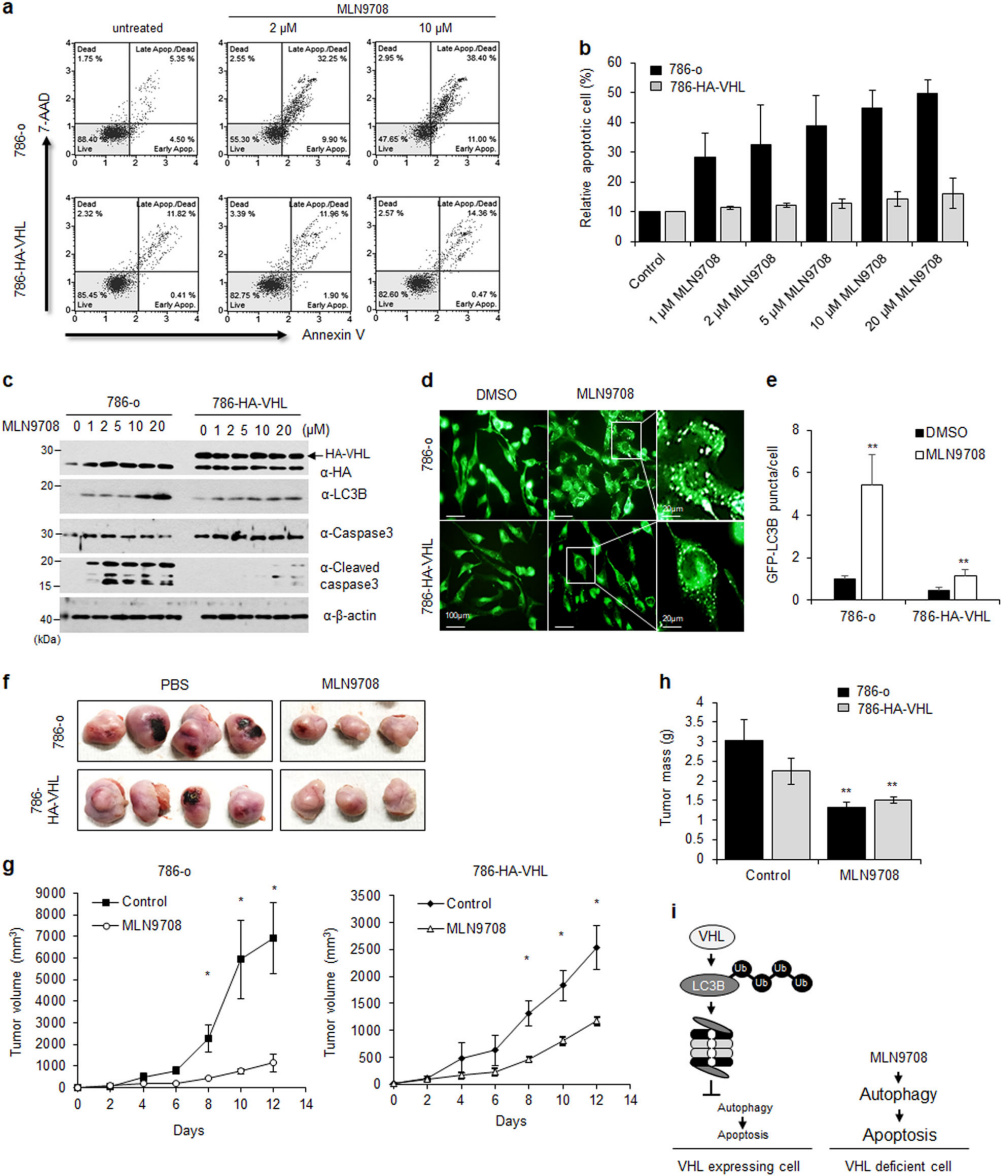
MLN9708 sensitizes VHL-defective cells more to cell death via autophagy signaling and represses tumorigenesis than wild-type VHL RCC cells. **a** Effect of MLN9708 on cell death was confirmed using flow cytometric analysis in 786-o or 786-HA-VHL cells after treatment with the indicated concentrations for 24 h. **b** The proportion of apoptotic cells was quantified using a Muse cell analyzer software and error bars represent the standard errors of means (±S.D.). **c** MLN9708-mediated autophagy and apoptosis signals were analyzed using western blotting in 786-o or 786-HA-VHL cells after treatment for 24 h. **d** The 786-o or 786-HA-VHL cells were transfected with GFP-tagged LC3B expression vector. After transfection for 24 h, the cells were treated with DMSO or 10 µM MLN9708 for 24 h, and aggregation of LC3B was observed using fluorescence microscopy. **e** The GFP-LC3B puncta in each cell (*N* = 10 cells/group) were quantified using the ImageJ software and the ratio of GFP-LC3B puncta per cell is represented as ±S.D. **f** Nodules were excised from mice 12 days after inoculation and photographed. **g** Tumor volume in mm^3^ was calculated using the formula volume = (length × width^2^)/2 using caliper measurements. **h** Tumor mass was measured at the end of the experiment. **i** Schematic diagram of VHL-mediated autophagy regulation

We selected a compound that elicited differences in cell death in 786-o and 786-HA-VHL cells treated with a library of autophagy-inducing compounds. MLN9708, a proteasome inhibitor, showed the most significant difference in the expression of LC3B. To confirm whether MLN9708 induces autophagy-dependent cell death in VHL-deficient RCC cells, 786-o and 786-HA-VHL cells were dose-dependently treated with MLN9708. Next, apoptosis was measured using annexin V and 7-AAD staining on a Muse cell analyzer. Results showed that apoptotic cell death of 786-o cells treated with MLN9708 was higher than that of 786-HA-VHL cells (Fig. 6a, b). After MLN9708 treatment of 786-o or 786-HA-VHL cells, the levels of autophagy- and apoptosis-related proteins were analyzed using western blotting. The 786-o cells showed induction of the LC3B-II form (a major marker of autophagy) and cleaved caspase-3 (a marker of apoptosis; Fig. 6c). To confirm whether MLN9708 activates caspase-3 via autophagic flux, the ATG5 KO cell line was treated with MLN9708 (Supplementary Fig. 7a). MLN9708 did not activate caspase-3 in ATG5 KO cells. Next, to identify the type of cell death induced by MLN9708, we analyzed the cell death in the MLN9708-treated VHL-deficient and VHL-expressing cell lines in the presence of the autophagy inhibitor, 3-MA, and the apoptosis inhibitor, Z-VAD-FMK. The inhibition of autophagy decreased cell death in VHL-deficient cells but not in VHL-expressing cells in the presence of 3-MA, whereas Z-VAD-FMK inhibited cell death in both cell lines (Supplementary Fig. 7). The cell death and the formation of LC3B puncta was increased in both VHL mutant (VHL-L101A) expressing cells and VHL-depleted cells upon treatment with MLN9708 (Supplementary Fig. 8a–d). Furthermore, the induction of active capase-3 was increased in the mutant VHL (L101A) expressing cell line same as VHL-deficient cells treated with MLN9708; 3-MA inhibited cell death significantly in both VHL-deficient and VHL-mutant expressing cells (Supplementary Fig. 8e–g). This result suggested that MLN9708 induced autophagy-related cell death in VHL-deficient cells. The LC3B puncta following increased LC3B expression were identified and quantified in 786-o or 786-HA-VHL cells after MLN9708 or dimethyl sulfoxide (DMSO) treatment. MLN9708-treated 786-o cells showed significant increase in the number of LC3B punctates than 786-HA-VHL cells (Fig. 6d, e). Based on these results, we concluded that the proteasome inhibitor, MLN9708, significantly activated autophagy in the VHL-deficient RCC cell line and caused cell death. To verify MNL9708 as a potential new drug candidate in VHL-deficient RCC, mice (5-week-old, female) were inoculated with 786-o or 786-HA-VHL cells. Mice were intravenously injected with MLN9708 or saline as negative control when the tumor volume reached 100 mm^3^. The length and width of tumors were measured after every 2 days. We observed that the proteasomal inhibitor MLN 9708 dramatically repressed tumor growth in VHL-deficient RCC (Fig. 6f, g). In addition, tumor mass was significantly reduced in VHL-deficient RCC than in VHL expressing RCC at end of the experiment (Fig. 6h). Figure 6i shows a schematic explaining regulation of autophagy and apoptosis by MLN9708 treatment in VHL-deficient or VHL expressing RCC cells. These results confirmed that MLN9708 is a potential anti-cancer drug for treating patients with VHL-deficient RCC.

## Discussion

Since the identification of VHL, efforts to identify its functions have been reported. The most important finding is probably its role as an E3 ubiquitin ligase, via which it degrades HIFs^8^. VHL inhibits the induction of various target molecules of HIFs, such as vascular endothelial growth factor (VEGF) and platelet-derived growth factor (PDGF), by forming a complex (BVC) with elongin B/C and Cullin2, thereby exerting its tumor suppressor effect^16^. HIF-independent functions of VHL, such as the regulation of cell cycle and intracellular microtubule stability, have been reported; however, no molecule has been identified as a new substrate for BVC. Interestingly, we observed that VHL-expressing cells are more resistant to environmental stress and maintained their original morphological features and viability when subjected to such stresses. In contrast, VHL-deficient cells easily formed intracellular vesicles and underwent apoptosis. These observations led us to hypothesize that VHL is involved in autophagy, which plays crucial role in cell homeostasis and death. Indeed, our results suggested that VHL downregulates autophagy, and, therefore, we aimed to determine the mechanism via which this happens.

LC3B is cleaved by ATG4 and then conjugated with phosphatidylethanolamine, which acts as a key component of autophagosomes^17^. Previous reports show that LC3B is activated in solid tumors and is associated with tumor progression^4,18^; however, the half-life of LC3B is not known. We showed that VHL interacted with LC3B via the LIR motif, which is located in the β-domain of VHL. Adaptor proteins such as p62 and NIX that harbor the LIR motif target cargo proteins to autophagosomes by interacting with LC3B^19^. Here, we provided evidence that VHL interacts and ubiquitinates LC3B, thereby downregulating LC3B expression. Previous studies have reported transcriptional regulation of LC3B by VHL via miR-204^20^. However, this is the first report showing that VHL degrades LC3B in a proteasome-dependent manner.

We used a renal cell carcinoma cell line, a well-established cell line for studying the role of VHL in regulating autophagy^21^. VHL loss-of-function is the most common cause for ccRCC. In VHL disease, VHL loss-of-function correlates strongly with RCC phenotype; however, this correlation is less clear in the sporadic disease^22^. HIF is stabilized in VHL-deficient cells, and thereby activates its target genes that are responsible for angiogenesis, metastasis, and invasion^23^. HIFs are also involved in regulating metabolism, autophagy, and adaptation to various cellular stresses in solid tumors^24^; however, the outcome of regulating autophagy, cell death, or survival depends on the cell type^25^. This might be explained by the heterogeneous environment of solid tumors, which show varied cellular context. Therefore, autophagy-based cancer therapy is a double-edged sword and must be applied after considering the cellular status.

Turcotte and colleagues claimed that STF-62247, an autophagy-inducing agent, selectively targets VHL-deficient cells^14^. In this study, we demonstrated that the proteasome inhibitor, MNL9708, induces cell death in a VHL-deficient cell line. MNL9708 has been developed for myeloma therapy^26^. The ubiquitin-dependent proteasome pathway and autophagy are two major related pathways for abnormal protein degradation. For example, the inhibition of proteasome induces autophagy and reduces autophagy promoted polyubiquitination^27^. Based on published reports and our data showing that VHL downregulates autophagy, we suggested that MLN9708 induced cell death in VHL-deficient cells due to excessive autophagy. Several proteasome inhibitors, including MNL9708, induced cell death more effectively in VHL-deficient cells than in VHL-expressing cells. In conclusion, proteasome inhibitors can be used for treating VHL-deficient RCC, as the tumor suppressor VHL targets LC3B in RCC, thereby regulating excessive autophagy-related cell death.

## Material and methods

### Antibodies and reagents

Antibodies against GST, His, 26S proteasome, ATG5, ATG7, ubiquitin, p62, LAMP1, VHL (Santa Cruz Biotechnology, Dallas, TX, USA), Flag, LC3B, β-actin (Sigma-Aldrich, St. Louis, MO, USA), caspase 3, cleaved caspase 3 (Cell Signaling, Danvers, MA, USA), HA (Abcam, James Staveley, VP), MDM2 (BD Pharmingen, San Jose, CA, USA), and HRP (horse radish peroxidase)-conjugated anti-mouse or rabbit IgG (Santa Cruz Biotechnology) were purchased. MG132 (proteasome inhibitor, #C2211), bafilomycin A1 (autolysosome inhibitor, #B1793), doxycycline (#D3447) and chloroquine (lysosome inhibitor, #C6628), 3-methyladenine (autophagy inhibitor, #M9281), and Z-VAD-FMK (apoptosis inhibitor, #V116) were purchased from Sigma-Aldrich. The autophagy compound libraries (156 small molecules) with autophagy inducing activity were purchased from Selleck Chemicals (Houston, TX, USA). Human RCC MTA blocks were purchased from US Biomax (Rockville, MD, USA). Antibody sources are listed in Supplementary Table S1.

### Plasmids

We generated plasmids encoding wild-type VHL, the various domains of VHL, and wild-type HIF-1a and ODD domain of HIF-1a for expression in bacterial cells or mammalian cells, as described in a previous study^15^. Mutants of VHL and LC3B were generated based on the wild-type genes using PCR. For bacterial expression, plasmids were constructed by ligating PCR products into pET28a (Novagen) and pGET4T-1 (Pharmacia). For mammalian expression, the coding region of LC3B was amplified from a HeLa cDNA library using the relevant primers and cloned into pFlag-CMV1 (Flag-tag, Sigma-Aldrich) and pEGFP-C2 (GFP-tag). The VHL-shRNA (5′-TGTTGACGGACAGCCTATT-3′) sequence was inserted into the pSUPER vector according to the manufacturer’s instructions (Invitrogen).

### Cell culture and transfections

The human renal carcinoma cell line 786-o, 786-o cells stably expressing wild-type VHL (786-HA-VHL), RCC, RCC cells stably expressing wild-type VHL (RCC-VHL), and human embryonic kidney cell line HEK293 were cultured at 37 °C in a humidified 5% CO_2_ atmosphere in Dulbecco’s modified Eagle’s medium (DMEM; Gibco BRL, Gaithersburg, MD, USA) supplemented with 10% fetal bovine serum (FBS, Gibco BRL), and antibiotic-antimycotic (Gibco BRL). HeLa cell line stably expressing EGFP-LC3 was transfected with the empty or pEGFP-LC3B expression vector and cultured in complete DMEM medium with 1 mg/ml G418 (GIBCO) for 1 month for single colony selection. The MEF-*Atg5* knockout cell line was a gift from Dr. Jung^28^. For *Atg5* knockout, the cells were cultured in complete DMEM with 20 ng/ml doxycycline hydrochloride (DOX, Sigma-Aldrich) for 5 days. The cells were transiently transfected using a standard calcium-phosphate method and Lipofectamine (Invitrogen, Carlsbad, CA, USA) according to the manufacturer’s protocol. The cells were counted using a hematocytometer.

### Reverse transcription-polymerase chain reaction

Total RNA was extracted using a TRIzol reagent-based kit (iNtRON Biotechnology). cDNA was synthesized using 1 μg of total RNA with the SuperScript III first strand synthesis system (Thermo Fisher Scientific) according to the manufacturer’s protocol. The cDNA was amplified using specific primers and quantified using a densitometry software. Data from independent experiments are shown as mean±standard deviation (S.D.). The primer sequences are listed in Supplementary Table S2.

### Western blot analysis

Cells were lysed in RIPA buffer (50 mM Tris-HCl, pH 8.0, 150 mM NaCl, 5 mM EDTA, and 0.1% SDS). The lysates were separated using sodium dodecyl sulfate-polyacrylamide gel electrophoresis (SDS-PAGE) and transferred to a polyvinylidene fluoride membrane (PVDF; EMD Millipore). The membranes were blocked with 5% skim milk in phosphate buffered saline Tween 20 (PBST) (0.05% Tween-20 in PBS) at room temperature (RT) for 1 h. The membranes were incubated with the specific primary antibodies in PBST at RT for 1 h or at 4 °C overnight, and then with secondary antibody in PBST at RT for 1 h. The proteins were detected using a chemiluminescence kit (Miracle-Star; iNtRON Biotechnology, Seoul, Korea and Immobilon; Millipore).

### Measurement of cell viability

The 786-o or 786-HA-VHL cells were seeded into a 96-well plate (5 × 10^3^ cells/well) and treated with 5 µM autophagy compound libraries for 24 h. Next, the cells were incubated in fresh complete DMEM and cell viability was assessed using the assay kit (LPS solution, Daejeon, Korea) according to the manufacturer’s protocol. Cell viability was determined by measuring optical density (OD) at 450 nm using a microplate reader (Molecular device, San Jose, CA, USA).

### Cytofluorimetric analysis of apoptosis and autophagy using the Muse™ cell analyzer

For confirming the apoptosis-inducing effect of the autophagy compound libraries on RCC cells, the cells seeded into a 24-well plate (5 × 10^4^ cells/well) were treated with 5 µM autophagy compound libraries for 24 h. Then, the cells were harvested, washed with ice-cold PBS, and re-suspended in complete DMEM. The cells (1 × 10^4^ cell/100 µl) were incubated with a Muse™ annexin V & dead cell assay kit (EMD Millipore, Hayward, CA, USA) at RT for 20 min and analyzed on a Muse™ cell analyzer (EMD Millipore). Data were quantified using the Muse™ analysis software (EMD Millipore). For autophagy detection in RCC cells after treatment with autophagy inducing compounds, the cells were treated with 5 µM autophagy compound libraries for 24 h. Subsequently, the cells were harvested, incubated with a Muse autophagy LC3-antibody based kit (EMD Millipore) according to the manufacturer’s protocol, and analyzed using a Muse™ cell analyzer.

### Flow-cytometric analysis

For apoptosis assay, 786-o, 786-VHL-WT, and 786-VHL-L101A cells treated with 10 µM MLN9708 for 18 h. The percentages of active caspase-3 positive cells were measured by flow cytometry to determine the level of apoptotic cell death. All analysis was performed using a BD Calibur with CELL-Quest software (BD Biosciences).

### Immunofluorescence staining

Cells were fixed with 4% paraformaldehyde for 15 min at 37 °C, followed by permeabilization with PBS containing 0.1% Triton X-100 for 15 min at RT. The cells were washed twice with PBS and incubated for 1 h at RT with 0.1% normal serum. Next, the cells were incubated overnight with anti-LC3B, anti-LAMP1, anti-VHL, and anti-20S proteasome primary antibodies at 4 °C. After the incubation, the cells were washed, incubated with goat-anti-mouse-Alexa488, goat-anti-mouse-Alexa594, or goat-anti-rabbit-Alexa488, and goat-anti-rabbit-Alexa594 secondary antibodies (Molecular Probes) for 1 h at RT, and observed using fluorescence microscopy (Olympus, Tokyo, Japan) and confocal microscopy (Zeiss, Oberkochen, Germany).

### Immunoprecipitation assay

In brief, cells were lysed in NET lysis buffer (20 mM Tris-Cl, pH 7.5, 100 mM NaCl, 1 mM EDTA, and 0.1% NP40 with proteinase inhibitor cocktail (Roche, Basel, Switzerland)), and incubated overnight with anti-Flag agarose beads (Sigma-Aldrich) at 4 °C. The antibody-antigen complex was washed in PBST and analyzed using western blotting. For the pull-down assay, the cell lysates or reaction mixtures were incubated with glutathione-Sepharose (GE Healthcare, Little Chalfont, UK) or Ni-NTA agarose beads (QIAGEN, Hilden, Germany) for 2 h at 4 °C with gentle shaking. After incubation, the beads were washed thrice with PBST. The bead-bound proteins were analyzed using western blotting.

### Purification of recombinant proteins from Escherichia coli

All 6× His-tagged proteins were expressed in *Escherichia coli* (*E. coli*) BL21 (DE3) cells and expression was induced using isopropyl β-D-1-thiogalactopyranoside (IPTG). Induced BL21 cells were harvested by centrifugation and the cells were lysed in buffer containing 20 mM Tris-HCl, 300 mM NaCl, 10 mM imidazole, and 1 mM PMSF, pH 7.5. After centrifugation, the cleared lysates were incubated with Ni-NTA agarose for 2 h at 4 °C. The bead-protein complexes were washed using buffer containing 20 mM Tris-HCl, 300 mM NaCl, and 20 mM imidazole, pH 7.5, and eluted in buffer containing 20 mM Tris-HCl and 250 mM imidazole, pH 7.5. For purifying GST-tagged recombinant proteins, the IPTG-induced cells were harvested and lysed in buffer (137 mM NaCl, 2.7 mM KCl, 10 mM Na_2_HPO_4_, 2 mM KH_2_PO_4_, 1 mM PMSF, pH 7.4) using sonication. After centrifugation, the cleared lysates were incubated with glutathione-Sepharose for 2 h at 4 °C. Bead-bound GST-fusion proteins were washed with PBS and eluted with a buffer containing 50 mM Tris, pH 8.8, 1 mM EDTA, 25 mM reduced glutathione, and 1 mM PMSF. All eluted proteins were dialyzed overnight in dialysis buffer (10 mM Tris-HCl, 50 mM NaCl, 10% glycerol, 0.5 mM DTT, 1 mM PMSF, pH 8.0) at 4 °C.

### In vitro and in vivo ubiquitination assays

For the in vitro ubiquitination assay, reaction mixtures were incubated with 0.2 µg E1 (Sigma-Aldrich), 0.5 µg GST-VHL/or His-VHL, 5 µg His-LC3B/or Flag-LC3B, 0.5 µg GST, and 25 μg/ml Flag-ubiquitin (Boston biochem) in reaction buffer [25 mM Tris-HCl, pH 7.5, 1 mM MgCl_2_, 2.5 mM DTT, 5 mM ATP, and the ATP regeneration system (1 mM creatin phosphatate, 1 mM creatin kinase, 0.5 μg/ml ubiquitin aldehyde)] at 37 °C for 1 h, followed by immunoprecipitation and analysis using western blotting. The S100 extracts were generated from 786-o cells based on previous reports^29^. The in vivo ubiquitination assay was performed as described previously^30^. In brief, cells were treated with MG132 for 16 h and lysed in NET buffer using sonication. After centrifugation, the cleared lysates were used for a pull-down assay and analyzed using western blotting.

### Mouse samples and in vivo xenograft assay

Five-week-old female BALB/c nu/nu mice were purchased from the Orient Bio (Seongnam, Korea) and maintained in accordance with the guidelines of the Institutional Review Committee for Animal Care and Use, KRIBB, under specific pathogen-free conditions.

The 786-o or 786-HA-VHL (5 × 10^6^ cells/in 100 µl PBS per mouse) cells were inoculated subcutaneously into the hindlimbs of mice. Mice were randomly segregated into treatment groups when the tumor was 100 mm^3^ in diameter. DMSO or MLN9708 (5 mg/kg body weight) was injected intravenously every 2 days for 12 days and tumor diameter was measured. Tumor volume was calculated as follows: tumor volume = (length × width)^2^/2. Tumor mass was measured at an end day of experiment.

### Statistical analysis

All data were repeated three or more times. Data represent mean ± S.D. Statistical significance of differences was assessed using the Student’s *t*-test.

## Supplementary information

Supplementary Tables

supplementary information
