# Supplementary material for: Ubiquitination of MAP1LC3B by pVHL is associated with autophagy and cell death in renal cell carcinoma
Source: Cell Death Dis. 2019 Mar 22;10(4):279. doi: 10.1038/s41419-019-1520-6 (PMC6430800; doi:10.1038/s41419-019-1520-6)
Supplement: Supplementary file 1 — Supplementary Tables [file 41419_2019_1520_MOESM1_ESM.docx]

**Supplementary Table S1. Antibodies sources**

| **Genes** | **Source** | **Host** | **Cat #** |
| --- | --- | --- | --- |
| Flag | Sigma-Aldrich | Mouse | F3165 |
| GST | Santa-cruz | Mouse | sc-53909 |
| His probe | Santa-cruz | Mouse | sc-8036 |
| HA probe | abcam | Mouse | ab-18181 |
| 26S proteasome α | Santa-cruz | Mouse | sc-65755 |
| ATG5 (Apg5) | Santa-cruz | Mouse | sc-133158 |
| ATG7 (Apg7) | Santa-cruz | Mouse | sc-33211 |
| P62 (SQSTM1) | Santa-cruz | Mouse | sc-28359 |
| LC3B | Sigma-Aldrich | Rabbit | L7543 |
| LAMP1 | Santa-cruz | Mouse | sc-20011 |
| Ubiquitin | Santa-cruz | Mouse | sc-8017 |
| VHL | Santa-cruz | Mouse | sc-135657 |
| MDM2 | BD pharmingen | Mouse | 556353 |
| Caspase3 | CST | Rabbit | 9662 |
| Cleaved caspase3 | CST | Rabbit | 9664 |
| β-actin | Santa-cruz | Mouse | sc-8432 |

**Supplementary Table S2. Primer sequences and conditions for conventional RT-PCR**

|  | **Genes**  **(Human)** | **Forward Primer (5’-3’)** | **Reverse Primer (5’-3’)** |
| --- | --- | --- | --- |
|  | *VHL* | cccaggtcatcttctgcaat | tcaatctcccatccgttgat |
| Autophagy-related genes | *LC3A* | ctcagaccggcctttcaa | ctcgtctttctcctgctcgt |
|  | *LC3B* | cggagaagaccttcaagcag | ctgggaggcatagaccatgt |
|  | *LC3C* | caagcagaggaaaagcttgg | gtctgtcctcaaggctgctc |
|  | *GABARAP* | agaagagcatccgttcgaga | cagaccgtagacactttcgtca |
|  | *GABARAPL1* | ggaggaccatccctttgagt | tcactgtaggccacatacagaa |
|  | *Gate16*  *(GABARAPL2)* | cactcgctggaacacagatg | gtgttctctccgctgtaggc |
|  | *P62* | gtggtaggaacccgctacaa | cacactctccccaacgttct |
|  | *Atg7* | acccagaagaagctgaacga | agacagagggcaggatagca |
|  | *Atg5* | agcaactctggatgggattg | cactgcagaggtgtttccaa |
|  | *b-actin* | agaaaatctggcaccacacc | ctccttaatgtcacgcacga |
