# Supplementary material for: Ubiquitination of MAP1LC3B by pVHL is associated with autophagy and cell death in renal cell carcinoma
Source: Cell Death Dis. 2019 Mar 22;10(4):279. doi: 10.1038/s41419-019-1520-6 (PMC6430800; doi:10.1038/s41419-019-1520-6)
Supplement: Supplementary file 2 — supplementary information [file 41419_2019_1520_MOESM2_ESM.docx]

**Ubiquitination of MAP1LC3B by pVHL is associated with autophagy and cell death in renal cell carcinoma**

Hyun Mi Kang^1^*, Kyung Hee Noh^1^*_,_ Tae Kyung Chang^1^, Dongmin Park^1^, Hyun-Soo Cho^1,2^, Jung Hwa Lim^1^ and Cho-Rok Jung^1,2^

^1^ Korea Research Institute of Bioscience and Biotechnology (KRIBB), 125 Gwahak-ro, Daejeon, Republic of Korea

^2^ Department of Functional Genomics, Korea University of Science and Technology (UST), 217 Gajeong-ro, Daejeon, Republic of Korea.

*_,_ These author contributed equally to this work.

**Running title**: Von Hippel-Lindau protein regulates macroautophagy

**Keywords**: Von Hippel-Lindau, microtubule-associated protein 1 light chain 3B, Ubiquitination, Autophagy, Renal cell carcinoma

Correspondence and requests for materials should be addressed to C.R. J. (crjung@kribb.re.kr) and J.H. L ([jhwa@kribb.re.kr](mailto:jhwa@kribb.re.kr)).

**Supplementary figure legends**

**Supplementary Fig. 1 RCC cells stably expressing VHL show low autophagy signaling under serum starvation condition.** (a) RCC cells or RCC cells stably expressing VHL were incubated in complete DMEM with 10% FBS or serum-free medium for 24 h and analyzed using western blotting with indicated antibodies. (b) Under the same condition as in Fig. S4a, the mRNA expression of autophagy-related genes was confirmed using RT-PCR.

**Supplementary Fig. 2 Autophagic flux is repressed in RCC cells stably expressing VHL after chloroquine treatment.** (a) Morphology changes of 786-o/786-HA-VHL or RCC/RCC-VHL cells without treatment or after treatment with 50 µM chloroquine (CQ) for 24 h using inverted phase contrast microscopy (with magnification of ×100 and ×250). The regions in black boxes show images with higher magnification. (b) Endogenous LC3B and LAMP1 expression was detected using immunofluorescence assay and fluorescence microscopy in 786-o or 786-HA-VHL cells after CQ treatment.

**Supplementary Fig. 3 E3 ligase activity of wild-type VHL and its various mutants** (a) HEK293 cells were transfected with 5 µg GST tagged wild-type VHL and its various mutants, and 5 µg Flag-HIF-1α plasmids. The cells were lysed, pulled down using GST Sepharose resin at 4°C for 2 h, and immunoblotted with the indicated antibodies. (b) *In vitro* HIF-ODD ubiquitination assay performed with 0.5 µg His-VHL and its various mutants, 5 µg GST-HIF-ODD (containing ODD domain within HIF-1a), and 5 µg GST (used as a negative control). After reaction for 1 h at 37°C, the reaction mixtures were pulled down with GST resin and immunoblotted.

**Supplementary Fig. 4 Ubiquitination of LC3B by VHL and its correlation with cell death.** (a) Endogenous VHL-depleted HeLa cells (siVHL-HeLa) were transfected with 5 µg of wild-type VHL or plasmids harboring various VHL mutants and harvested at 24 h post-transfection. The expression of indicated proteins was analyzed by immunoblotting using specific antibodies. (b) siVHL-HeLa cells were transfected with 5 µg of wild-type VHL or plasmids harboring various VHL mutants and cultured in complete medium (10% FBS) or serum-free medium (0% FBS) for 24 h. The harvested cells were counted using a hematocytometer.

**Supplementary Fig. 5** **Screening apoptotic cell death with autophagy modulators in the VHL-defective or stably VHL expressing RCC cell line.** (a) The 786-o or 786-HA-VHL cells were treated with a library of autophagy modulators, followed by staining using Muse annexin V and dead cell assay kit and analysis on the Muse cell analyzer. (b) The proportion of early and late apoptotic cells was represented graphically. Error bars represent ± standard deviation (S.D.)

**Supplementary Fig. 6** **Screening of autophagy induction by autophagy modulators in the VHL-defective or stably VHL-expressing RCC cell lines.** (a) Autophagy was measured using a Muse cell analyzer and the Muse autophagy LC3 antibody-based kit. The 786-o or 786-HA-VHL cells were not treated or treated with a library of autophagy modulators at 37°C for 24 h. The gray histogram represents control sample fluorescence and the red histogram represents test sample fluorescence. (b) The autophagy induction ratio was quantified using the Muse cell analyzer software. Error bar represents ± standard deviation (S.D.)

**Supplementary Fig. 7. Autophagy-related cell death and MNL9708.** (a) The *Atg5* knockout MEFs were left untreated or were treated with MNL9708 for 24 h and then analyzed using western blotting with the indicated antibodies. (b) The 786-o or 786-HA-VHL cells were treated with 3-MA (5 mM) for 4 h for 12 h and then with MLN9708 (5 µM) for 24 h. The treated cells were stained using Muse annexin V and a dead cell assay kit and then analyzed using the Muse cell analyzer. (c) The apoptosis induction ratio was quantified using the Muse cell analyzer software. Error bar represents ± standard deviation (S.D.) (d) Z-VAD-FMK (20 µM) for 12 h and then with MLN9708 (5 µM) for 24 h. The treated cells were stained using Muse annexin V and a dead cell assay kit and then analyzed using the Muse cell analyzer. (e) The apoptosis induction ratio was quantified using the Muse cell analyzer software. Error bar represents ± standard deviation (S.D.)

**Supplementary Fig. 8 MNL9708 induced cell death and the function of VHL.** (a) The 786-o, 786-HA-VHL, and 786-VHL mutant (L101A) cell lines were treated with 3-MA (5 mM) for 4 h or Z-VAD-FMK (20 µM) for 12 h and then treated with MLN9708 (10 µM) for 18 h. The treated cells were stained and then analyzed using the Muse cell analyzer. (b) The apoptosis induction ratio was quantified using the Muse cell analyzer software. Error bar represents ± standard deviation (S.D.) (c) GFP-LC3 vector was transfected with 786-o, 786-VHL WT, and 786-VHL L101A expressing cells. The cells were treated with 10 μM MNL9708 for 18 h and then GFP-LC3 puncta were identified using fluorescence microscopy. (d) The induction of LC3B puncta cell ratio was quantified by counting. Error bar represents ± standard deviation (S.D.) (e) 786-o, 786-VHL-WT and 786-VHL-L101A cells treated with MLN9708 (10 µM) for 18hr. The expression of indicated proteins was analyzed by immunoblotting using specific antibodies. (f)(g) The 786-o, 786-HA-VHL, and 786-VHL mutant (L101A) cell lines were treated with 3-MA (5 mM) for 4 h or Z-VAD-FMK (20 µM) for 12 h and then treated with MLN-9708 (10 µM) for 18 h. Cell apoptosis (caspase based) was analyzed using a FACS Calibur system. The active capase-3 induction ration was quantified using the Muse cell analyzer software. Error bar represents ± standard deviation (S.D.)


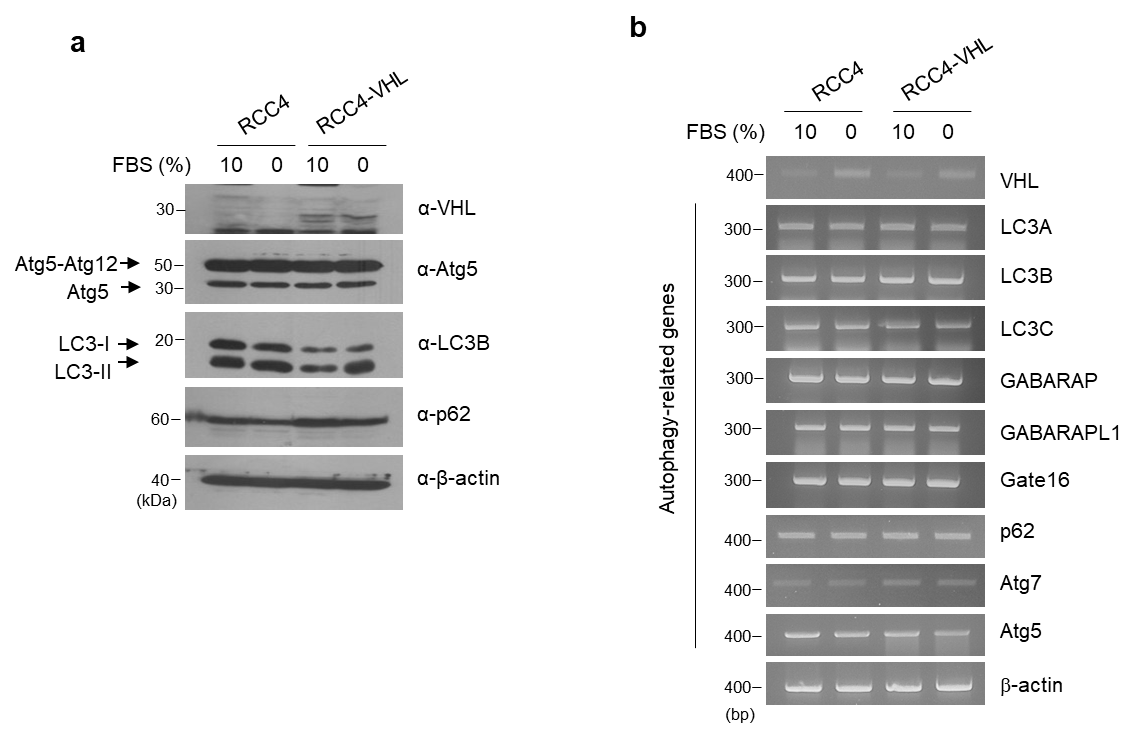


**Supplementary Fig. 1**

**
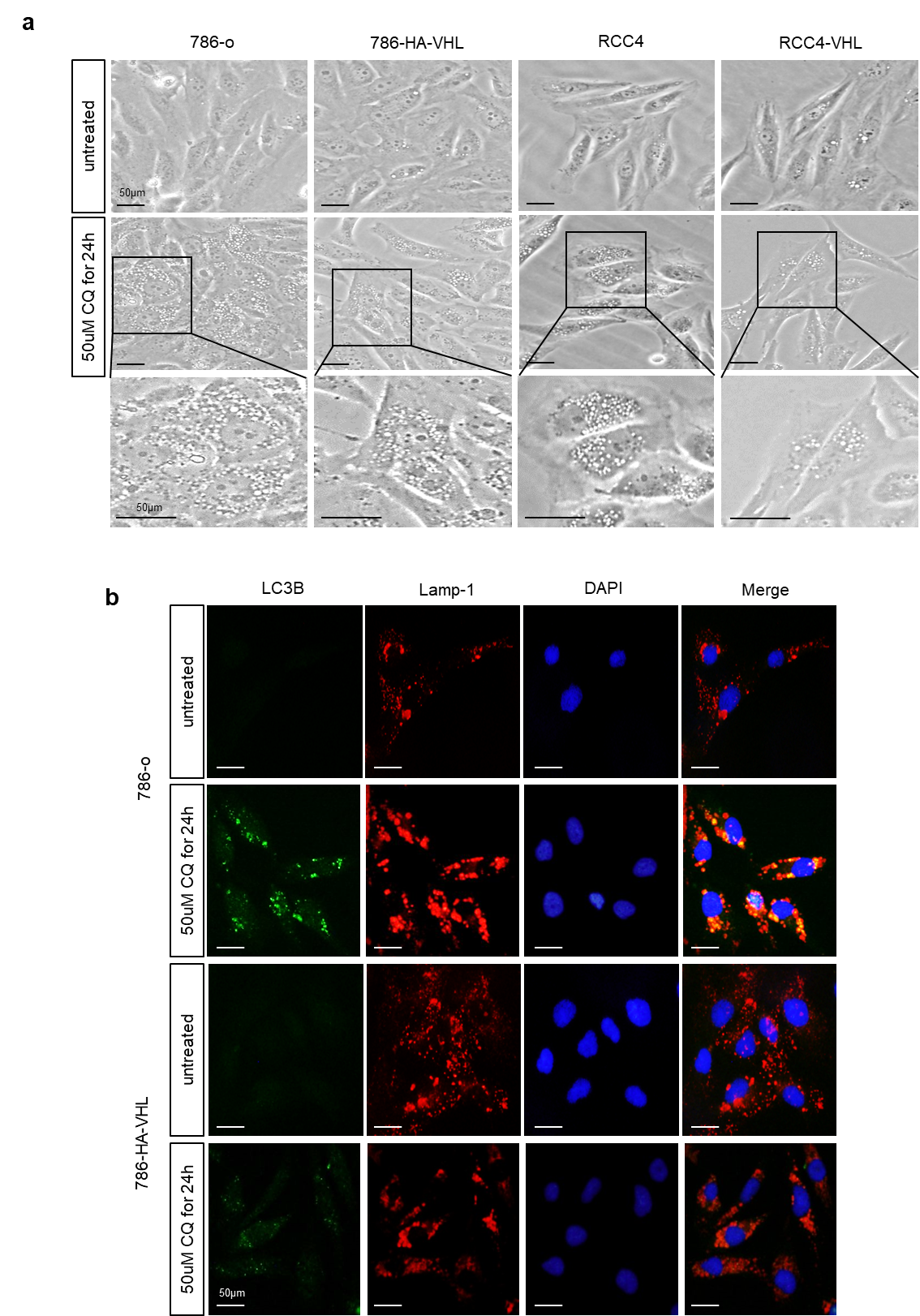
**

**Supplementary Fig. 2**

**
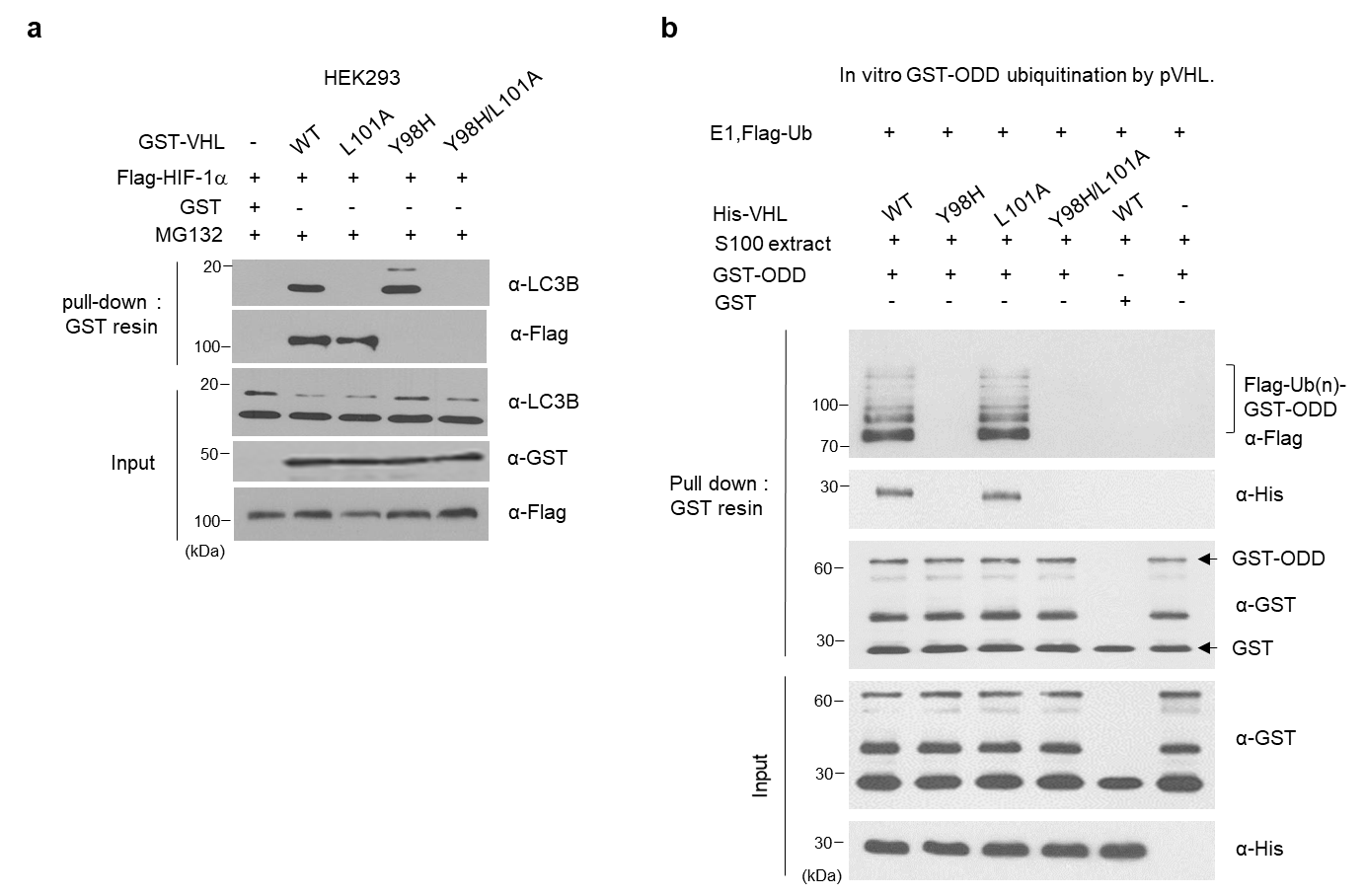
**

**Supplementary Fig. 3**

**
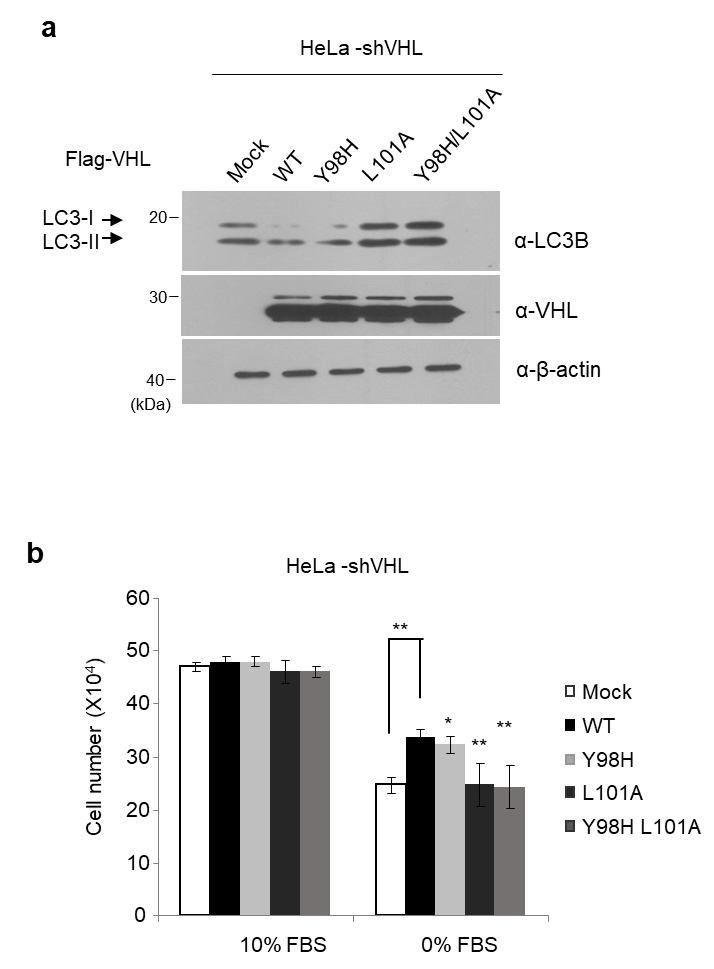
**

**Supplementary Fig. 4**


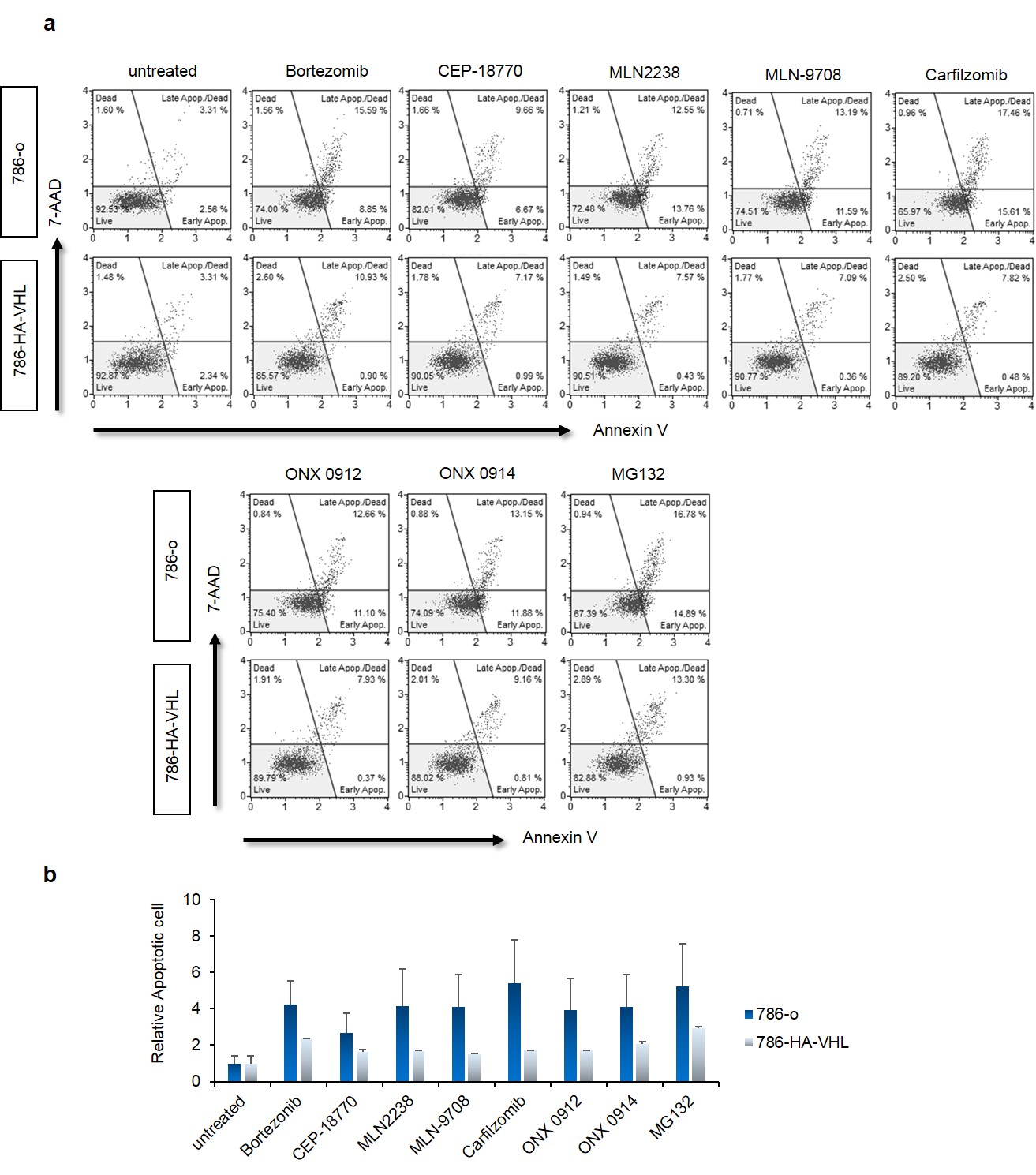


**Supplementary Fig. 5**

**
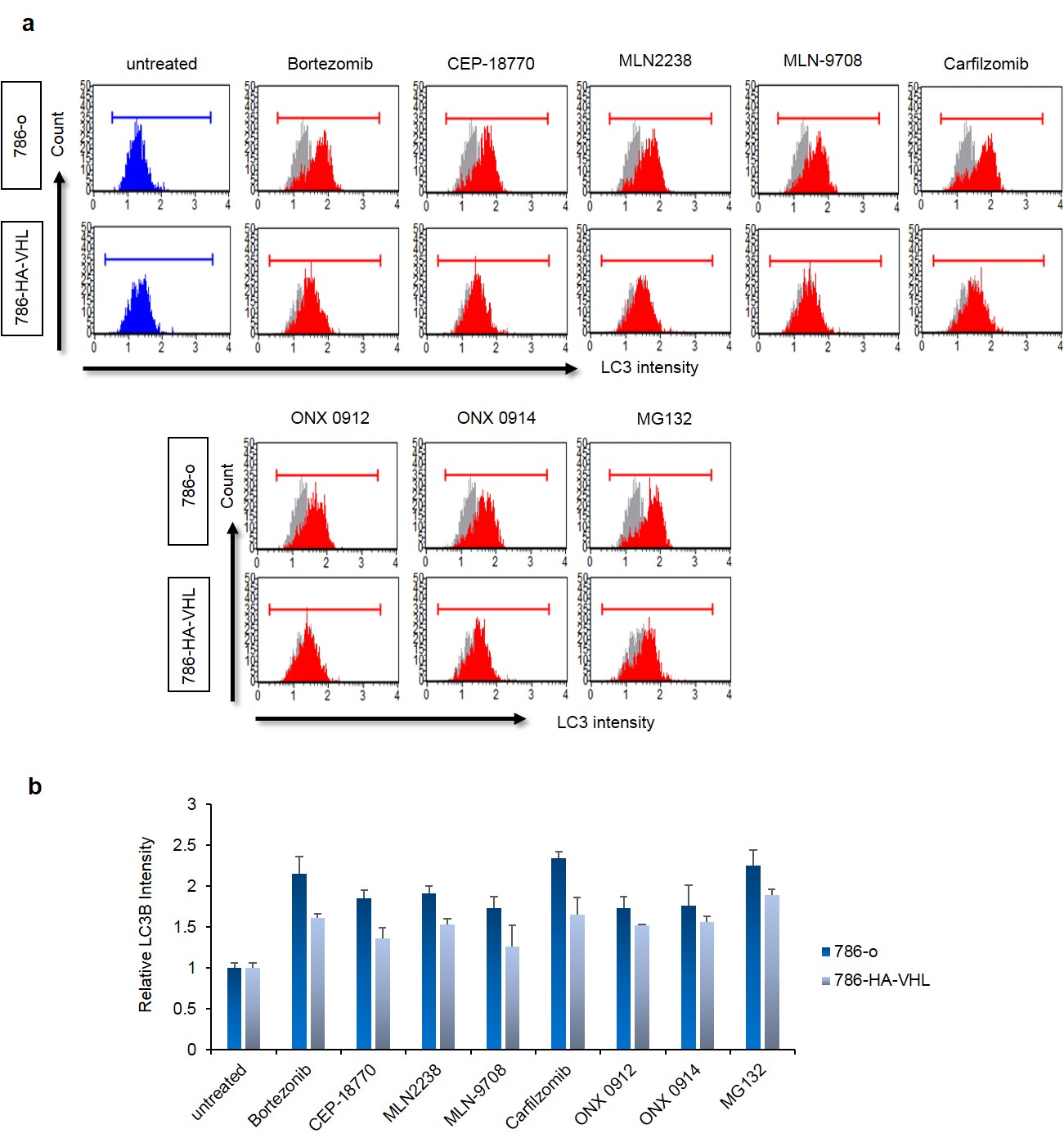
**

**Supplementary Fig. 6**

**
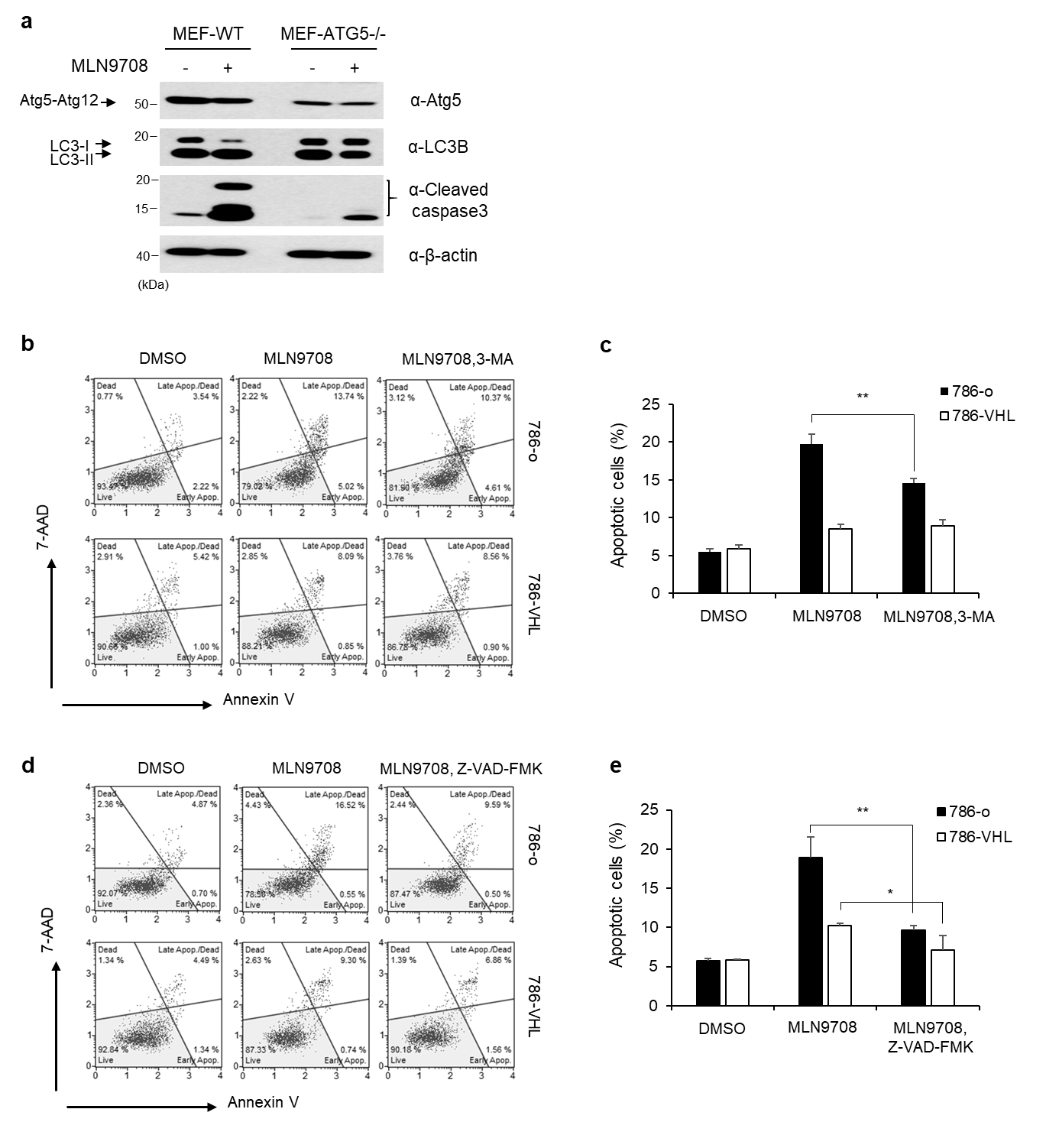
**

**Supplementary Fig. 7**

**
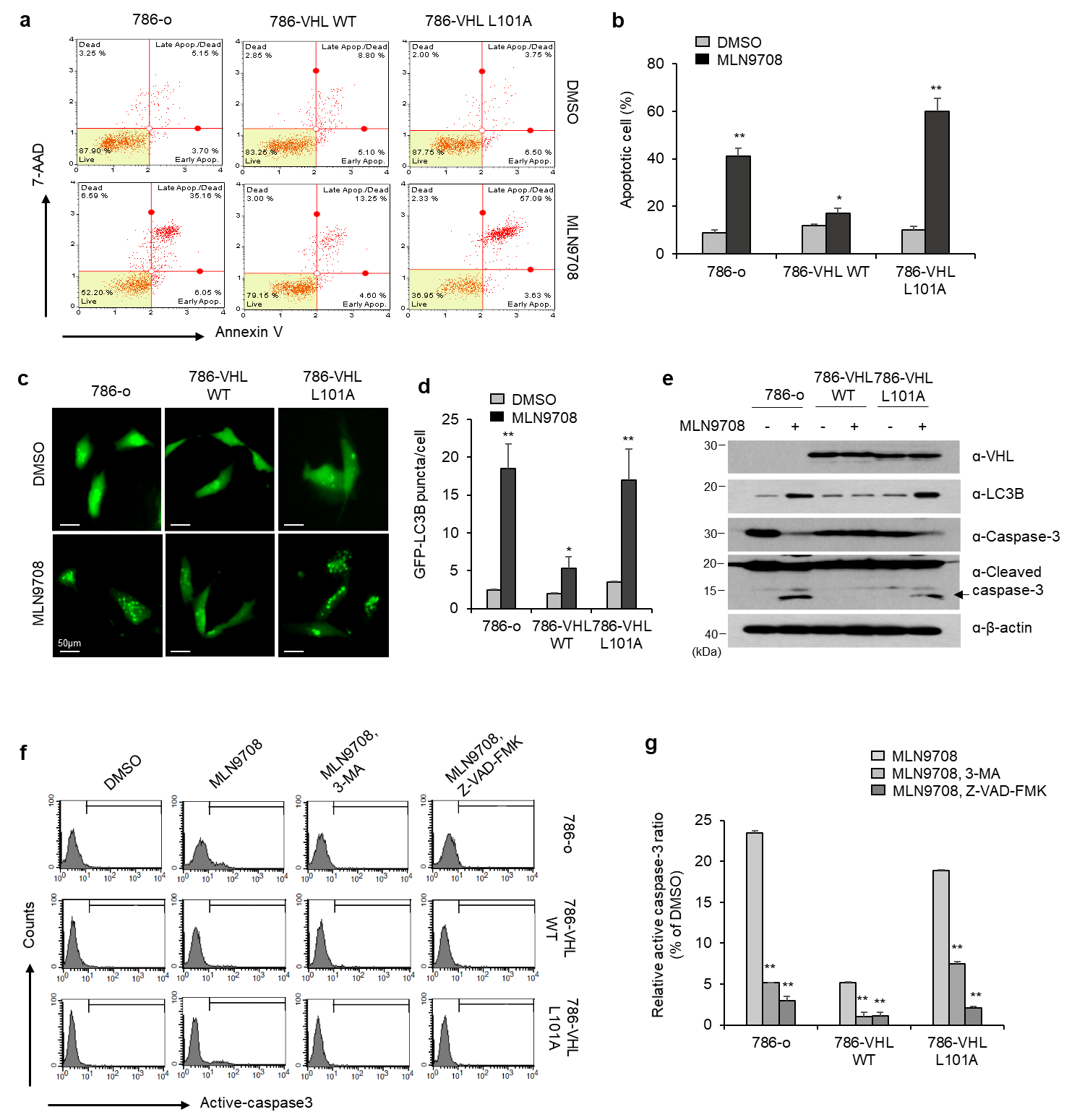
**

**Supplementary Fig. 8**
